# Supplementary material for: Genetic engineering of Staphylococcus haemolyticus: overcoming restriction-modification barriers and targeting virulence genes
Source: Microb Genom. 2026 Jul 29;12(7):001780. doi: 10.1099/mgen.0.001780 (PMC13417997; doi:10.1099/mgen.0.001780)
Supplement: Supplementary Material 1. [file mgen-12-01780-s001.pdf]

Supplementary Methods:

Genomic DNA isolation for high-purity, high molecular weight DNA

High molecular weight (HMW) genomic DNA was isolated using a modified protocol based on the MasterPure™ Gram Positive DNA Purification Kit (Lucigen). Bacteria were grown overnight in 10 ml of TSB, at 37°C, with shaking at 250 rpm. The overnight cultures were pelleted by centrifugation at 5000 rpm for 10 minutes. The supernatant was removed and the tubes were inverted onto tissue paper to draw out residual media. Resuspend the cells in at least 10 ml TES buffer (20 mM Tris-HCl, pH 8.0, and 50 mM EDTA) and normalize the sample to OD600 of 1. 10 ml of the normalized cell suspension were transferred to a new centrifuge tube and pelleted at 5000rpm for 10min. The supernatant was removed and the cells resuspended in 300 µL of TE buffer (from the kit). The suspension was transferred to Lysing Matrix B tubes, and vortexed briefly before 1 µL of lysozyme (Ready-Lyse Lysozyme Solution,~ 30,000 U/µL) was added. Samples were vortexed briefly, pulse-spun, and incubated at 37°C for 20 minutes. Following this, 300 µL of GP lysis solution was added, and the samples were bead-beaten at 6.0 m/s for 20 seconds using an MP FastPrep®-24 instrument. For tougher strains, additional bead-beat cycles may be performed with 5-minute cooling intervals on ice.

Proteinase K (2 µL) was added to each sample, followed by incubation at 65°C for 15 minutes. Samples were cooled to 37°C for 5 minutes, placed on ice for 3–5 minutes. 350 µL of MPC Protein Precipitation Reagent was added, then mixed by pulse vortexing for 3-5 seconds before placing back on ice. Debris was pelleted by centrifugation at 10 000 g for 10 minutes at 4°C. The supernatant was carefully transferred to a clean1.5 ml Eppendorf tube, centrifuged again for 2 minutes at 10 000 *g*, and transferred to a new tube to minimize debris carryover. RNase A (2 µL) was added, and samples were incubated at 37°C for 30 minutes.

DNA was precipitated by mixing the samples with 700 µL of DNA-grade isopropanol, inverting 30–40 times, and centrifuging at 10,000 rpm for 10 minutes at 4°C. The resulting DNA pellet was washed twice with 1 ml of 70% ethanol (the pellet was carefully dislodged from the side of the tube by gentle pipetting or flicking the tube, then the tube was inverted several times to ensure removal of the salts). The ethanol was removed, and the tubes were briefly centrifuged before the remaining ethanol was removed by pipette. The pellets were air-dried, then resuspended in 50–200 µL of pre-warmed TE buffer or water at 37°C. If the pellets were tricky to resuspend, the tubes were incubated at 37°C in a dry bath until they became translucent and flicked gently.

DNA quality was assessed using a Nanodrop spectrophotometer (A260/280 and A260/230 ratios), a Qubit fluorometer for dsDNA quantification, and agarose gel electrophoresis to confirm HMW integrity. Only DNA with a yield of 10–15 µg and minimal fragmentation was used for PacBio sequencing.

Syngenic DNA

Isolate 51-13 has the methylation motifs GAGG/CCTC (Type II) and CRTANNNNNNNTTC (Type I). We used plasmid pEPSA5, which had previously been modified to have minicircle-forming properties(1). pEPSA5 did not contain any CRTANNNNNNNTTC motifs, but did have 25 GAGG/CCTC motifs, 17 of which were located on the *E. coli* replicon and were not edited because they would be removed during minicircle formation. The remaining 8 motifs were located in the *S. aureus* replicon. We edited the plasmid sequence using DNAstar. When the motifs fell within coding sequences, we aimed to create silent mutations that

would not change the amino acid codon in that position. Two motifs were located within the promoter region of the chloramphenicol resistance gene; these we left unaltered. These edited sequences were sent to Synbio Technologies (Monmouth Junction, USA) to be synthesised. The synthesised fragments and plasmid backbone were used as templates for PCR in which primers contained overlaps needed for assembling the fragments into circular plasmids. After cleaning the PCR products and denaturing template DNA using DpnI (New England Biolabs, USA), we used NEBuilder Hi-Fi Assembly Master Mix (New England BioLabs, USA) to assemble the plasmids. Assembled plasmids were transformed into NEB 5-alpha Competent *E. coli*. Colony PCR (see supplementary table 1 for primers) was done the following day to select correctly assembled plasmids, which were isolated using the NucleoSpin Plasmid kit (Macherey Nagel, country). The minicircles were linearised with restriction enzyme HindIII (New England BioLabs, USA) to check for residual full plasmids.

Table S8. Composition of the plasmids and minicircles constructed for transformation of S. haemolyticus isolate 51-13. The suffix “Syn” denotes a syngenic construct in which GAGG sites have been edited out, while the “MC” suffix denotes a minicircle, which is induced to lose the *E. coli* replicon borne on pEPSA5.

Construct	Size (bp)	Number of GAGG sites
pEPSA5	8361	25
pEPSA5MiniMC	2668	8
pEPSA5MiniSynMC	2668	2

Simplified *E. coli* transformation

Isolated plasmids were electroporated into JMC1, an *E. coli* strain previously modified to produce minicircles (1, 2). A simplified electroporation protocol was used (3): JMC1 was grown on LB agar at 37°C overnight. A small amount of culture (about 2 µL) was picked off the plate with a sterile 1 µL inoculation loop and placed into 700 µL of sterile Milli-Q water in a 1.5 ml Eppendorf tube, then vortexed to resuspend. The cells were pelleted at 8000 *g* for 3 minutes; then the supernatant was removed. The cells were resuspended in 700 µL of sterile Milli-Q water and pelleted again at 8000 *g* for 3 minutes. The supernatant was removed, and the cells were resuspended in 40 µL of sterile Milli-Q water. The plasmids or minicircles were added (approximately 100 ng) to the cells and mixed gently before being transferred to a 0.1 cm electroporation cuvette (Biorad). The cells were electroporated at 1.8 kV, 200 Ω and 25 µF (Biorad Gene Pulser Xcell Microbial System). After electroporation, 1 ml of pre-warmed LB broth was added to the cells before transfer to a fresh Eppendorf tube. The cells were incubated at 37°C with gentle shaking for 1 hour before being plated out on LB agar containing 50 µg/ml kanamycin and incubated overnight. Colony PCR was done to confirm the presence of the plasmid/minicircle.

Minicircle induction

A single transformant colony was selected and used to inoculate 5 ml Terrific Broth (TB) containing 50 µg/ml kanamycin and incubated with shaking at 250 rpm at 30°C for 4-6 hours. This pre-culture was diluted 1:200 in fresh TB broth + 50 µg/ml kanamycin in an Erlenmeyer flask (5 × the volume of the broth), and incubated with shaking at 250 rpm at 30°C overnight (but not more than 16 hours).

The pH and OD600 of the overnight culture were measured. If the OD600 is > 8 with pH lower than 6,5, check the ventilation of the incubator and start over. If the pH was above 6.5, and the OD600 was between 4 and 6, the overnight culture was combined with the same volume of induction media, and if the OD600 was between 6 and 8, the volume of induction media added was double that of the overnight culture. Induction media was made up of 400 ml LB broth, 16 ml 1N NaOH and 400 µl 20% Arabinose, filter sterilised. A 1 ml aliquot (time 0, pre-induction) was removed for plasmid isolation. The induction mix was then incubated at 30°C with shaking at 250 rpm for 3 hours, and then at 37°C for 1 hour. Another 1 ml aliquot was removed for plasmid isolation and run on a gel alongside the time 0 pre-induction plasmid. If induction was not complete, the flask was returned to the 30°C and incubated with shaking for another hour. When induction was completed, the bacteria were pelleted by centrifuging at 5000 *g* for 10 minutes. The supernatant was removed, and the pellets either underwent minicircle isolation immediately using the NucleoSpin Plasmid kit (maxi prep), or they were frozen at -20°C until minicircle isolation could be done.

The full-sized plasmids and minicircles were used to optimise the transformation of *S. haemolyticus* 51-13 by tweaking the conditions used to produce competent cells, electroporation conditions, and recovery conditions.

***S. haemolyticus* competent cell preparation and electroporation**

For preparing competent cells, a saturated overnight culture of *S. haemolyticus* was diluted to an OD600 of 0.25 with TSB and incubated in an Erlenmeyer flask (5x the volume of broth used) at 37°C, with shaking at 250 rpm. When the OD600 reached 0.8-1.0, the flask was placed in an ice slurry for 15 minutes. The cells were harvested by centrifuging at 5000 *g* for 10 minutes at 4°C (all centrifuging was done at 4°C). The cells were washed in an equal volume of ice-cold ddH$_2$O, then pelleted again at 5000 *g* for 10 minutes. The wash step was repeated, then after pelleting, the cells were resuspended in 1/10 volume of 10% glycerol (ice cold). The cells were pelleted again, then resuspended in 1/25 volume 10% glycerol. The cells were concentrated in this stepwise fashion using 1/50 and then 1/100 volumes. Finally, the cells were resuspended in 1/165 volume and aliquoted into suitable volumes and frozen at -80°C until use.

For electroporation, the aliquots were thawed on ice (5 minutes) and then on the bench (5 minutes). The cells were pelleted by centrifuging at 5000 *g* for 1 minute, followed by removal of the supernatant. The cells were resuspended in the same volume of 10% glycerol + 0.5 M sucrose and split into 50 µL aliquots in 1.5 ml microcentrifuge tubes. 1-5 µg of the plasmid/minicircle was added and mixed gently by pipetting up and down 2-3 times, before incubation on the bench for 10 minutes. The plasmid and cell mix was transferred to a 1 mm electroporation cuvette (Biorad) and pulsed at 2,5 V, 100 Ω, 25 µF (Biorad Gene Pulser Xcell Microbial System). 950 µL of recovery buffer (TSB + 0.5 M sucrose) was added, and the cells were incubated at 37°C with gentle rotation (250-300 rpm) for 1 hour before plating the bacteria out on selective media and incubating for 24-48 hours (depending on the plasmid used).

Constructing JMC4

Assessing our *S. haemolyticus* collection, we found that 55 isolates had a methylation system identical to the one in 51-13 that produced the GAGG/CCTC motif. This, in combination with the tendency of the

GAGG/CCTC motif to fall within regulatory regions (making it difficult to edit out), led to our decision to construct an *E. coli* strain to replicate the GAGG/CCTC pattern in passaged plasmids. We used the method described by Jiang, Chen (4) to knock the *S. haemolyticus* MTase gene into JMC1 *E. coli*, while simultaneously knocking out the native *dcm* gene (a MTase methylating the CCWGG motif).

We constructed the editing template by using primer set in Table S1 to amplify the MTase gene in 51-13, and to amplify the upstream and downstream regions of the *dcm* gene from JMC1. The three sections were assembled into plasmid pRRS using the NEBuilder Hi-Fi Assembly Master Mix, with the MTase gene positioned between the two *E. coli* flanking regions. This construct (editing template) was linearized by PCR.

JMC1 was transformed with the pCas plasmid, which contained the λ-Red genes in addition to the Cas9 gene. JMC1+pCas electroporated with 100 ng of plasmid pTarget (obtained from Johnston et al, already carrying the sgRNA targeting dcm) and 400 ng of the editing template. After recovery at 30°C, the bacteria were plated out on LB agar containing kanamycin and spectinomycin and incubated at 30°C overnight. Confirmation of the insertion was determined by PCR and whole genome sequencing (sequencing and methylation analysis done in-house by the Johnston lab). DNA from the transformants was also digested with restriction enzyme MnII to ascertain whether the GAGG/CCTC motif was successfully methylated (see supplementary results 1). The resulting strain was named JMC4, and it is able to replicate the GAGG/CCTC methylation pattern and produce minicircles.

Hybrid approach

It is possible to combine the mimicry and SynGenic strategies. When plasmids were passaged through JMC4, it was not necessary to edit out the GAGG motifs. However, some plasmids contained motifs of type I systems. Since the motifs did not occur many times on any of the plasmids, we employed site-directed mutagenesis instead of DNA synthesis to edit out these motifs. Plasmids were amplified using primers designed to replace individual nucleotides in order to disrupt the type I restriction target sequence. Amplification was done using Q5 2X Mastermix, and the product was used as a template for a KLD reaction (New England BioLabs), resulting in a re-circularised plasmid without the type I system motifs. The plasmids were then passaged through JMC4 before being transformed into the target strains.

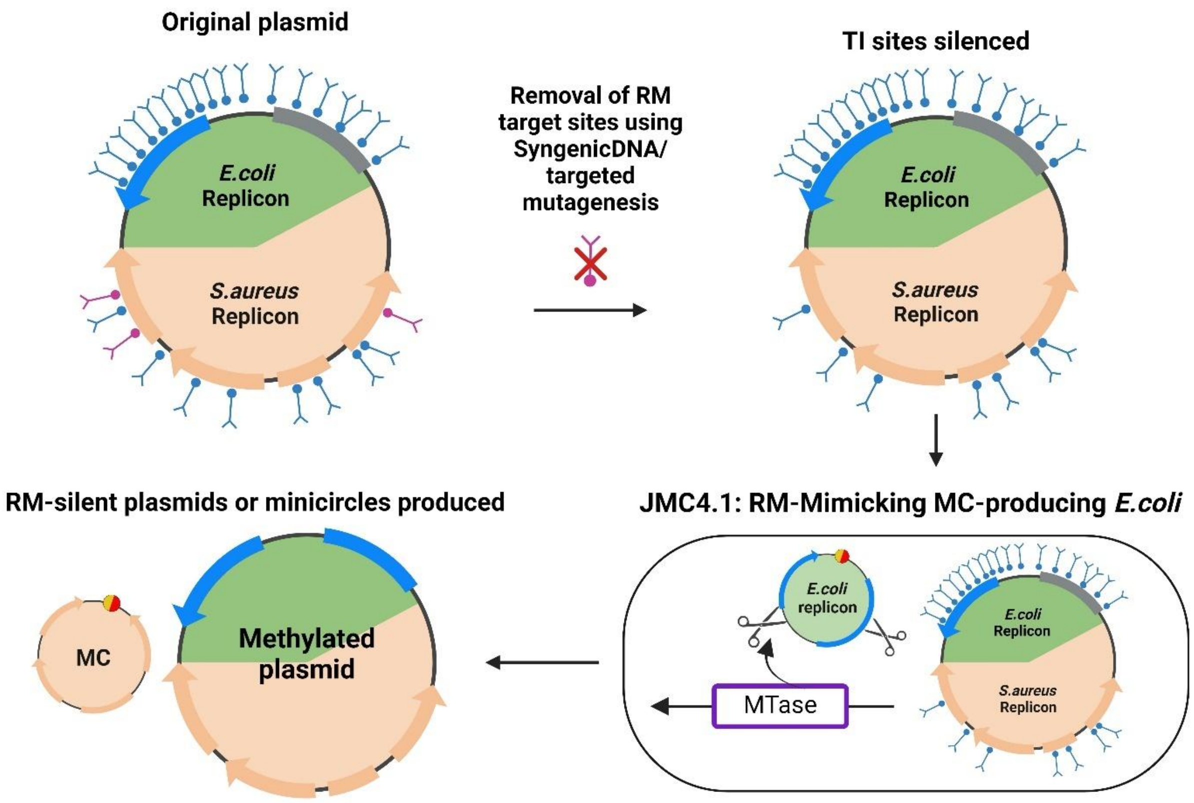

Figure S5: Combining mimicry and Syngenic strategies to create RM-silent plasmids for transformation into *S. haemolyticus*

Allelic exchange

Plasmid pIMAY-Z carried chloramphenicol as a resistance marker and the LacZ gene which allows for phenotypic screening of plasmid integration and excision. It works by allelic exchange, which uses homologous recombination to incorporate a template that excludes the gene to be deleted. We targeted several genes in strain 53-38 for deletion: *sraP*, *secA2*, *capA* and *capA*. We also replaced the capsule operon with the genes *EpSN* and *tagU*.

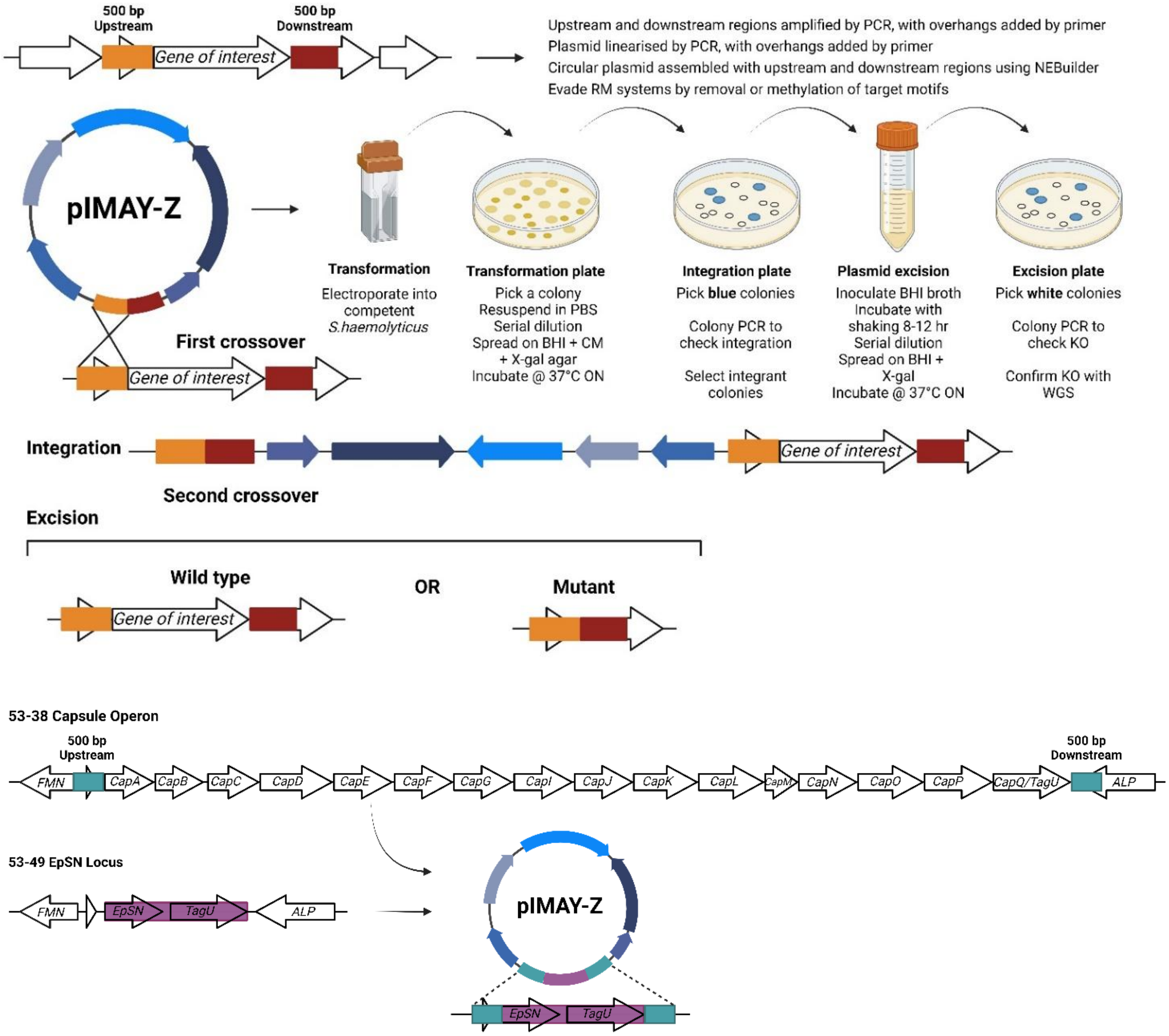

Figure S6: Summary of the allelic exchange process and construction of the insert for simultaneous deletion of capsule operon and insertion of the *EpSN* and *TagU* genes.

We followed the method set out in Monk and Stinear (5), except for the following changes: We used the NEBuilder Hi-Fi Assembly Master Mix to assemble the plasmid, instead of SLiCE, and adapted the methods for producing competent cells and transformation for *S. haemolyticus* (described above). Type I restriction sites on the plasmids were removed using site-directed mutagenesis, and plasmids were passaged through JMC4.1 to mimic GAGG/CCTC methylation.

For each of the genes of interest, a version of the pIMAY-Z plasmid containing a template for allelic exchange was constructed using NEBuilder® HiFi DNA Assembly. In each case, the recombination template was made by selecting approximately 500 bp upstream and downstream of the gene of interest, amplifying them by PCR, using Q5® High-Fidelity 2X Master Mix (New England Biolabs) and genomic DNA from 53-38 as a template. The plasmid was linearized by PCR. The primers belonging to fragments which

would be adjacent to each other when the vector was assembled contained matching overlapping sequences at their 5' ends.

In non-capsulated *S. haemolyticus* isolates, a different gene *EpSN*, as well as *tagU* are present in the locus where the capsule operon is found in capsulated strains. For this experiment, instead of deleting the capsule operon, the operon was replaced by the *EpSN* gene and the *TagU* cell envelope-associated transcriptional attenuator gene which was located immediately downstream. This region was amplified from 53-49, a non-capsulated isolate. The regions upstream and downstream of the capsule operon in isolate 53-38 were also amplified, and all three fragments were assembled into the pIMAY-Z plasmid. The plasmid was then treated in the same manner as the other allelic exchange vectors described above.

We did not obtain any direct integrants, so followed the slow integration protocol following transformation (5, 6), as illustrated in figure S6. Briefly, a colony which appeared on BHI + CM plates after 24-48 hr incubation at 30°C was picked and homogenized in PBS, then serially diluted up to 10^{-6} before spreading on BHI agar + CM + X-gal. Blue colonies indicated plasmid integration. Several blue colonies were examined using colony PCR to determine the side of plasmid integration. To excise the plasmids, a selection of these colonies (representing both sides of integration, if possible) was then inoculated individually into 10 ml of BHI broth and incubated with shaking until saturation. We serially diluted the saturated broth and plated out the 10^{-4}-10^{-6} dilutions onto BHI agar + Xgal and incubated the plates overnight at 37°C. White colonies were picked and reinoculated on fresh BHI + Xgal plates as well as BHI + CM and incubated overnight at 37°C. Colonies which remained white on BHI + Xgal and were unable to grow on BHI + CM were examined by colony PCR to determine whether the recombination template had been successfully integrated. Colonies with the expected amplicon size (indicating KO of target gene) were kept. Genomic DNA was isolated from these colonies and sent for WGS (Azenta).

References

1. Johnston CD, Cotton SL, Rittling SR, Starr JR, Borisy GG, Dewhirst FE, Lemon KP. Systematic evasion of the restriction-modification barrier in bacteria. Proceedings of the National Academy of Sciences. 2019;116(23), 10.1073/pnas.1820256116.
2. Johnston C, Cotton S. Minicircle producing bacteria engineered to differentially methylate nucleic acid molecules therein. Google Patents; 2022.
3. Granato E, Wittbrodt A. Quick'n'Dirty electrocompetent *E. coli* cells. 2020,
4. Jiang Y, Chen B, Duan C, Sun B, Yang J, Yang S. Multigene editing in the *Escherichia coli* genome via the CRISPR-Cas9 system. Appl Environ Microbiol. 2015;81(7), 10.1128/AEM.04023-14.
5. Monk IR, Stinear TP. From cloning to mutant in 5 days: rapid allelic exchange in *Staphylococcus aureus*. Access Microbiology. 2021;3(2),
6. Monk IR, Foster TJ. Genetic manipulation of Staphylococci—breaking through the barrier. Frontiers in cellular and infection microbiology. 2012;2, ARTN 4910.3389/fcimb.2012.00049.

Supplementary Figures

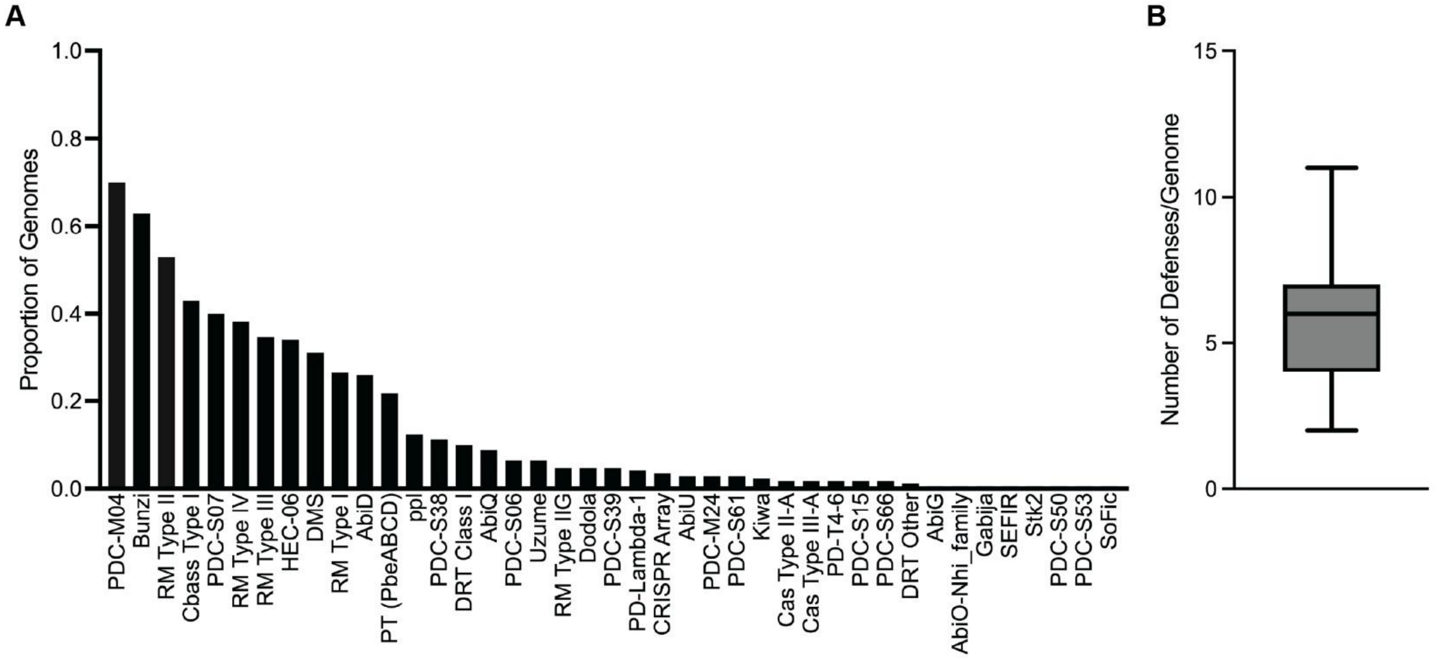

Figure S1) Number of genomes which contain each of the defense systems detected (**A**), and number of defense systems per genome (**B**). PDC, Phage defence candidate; RM, restriction modification; Cbass, cyclic-oligonucleotide-based antiphage signalling system; Hec, Hma-embedded candidate; DMS, DNA-modification system; Abi, abortive infection; PT, Phosphorotioation system; ppl, polymerase/histidinol phosphatase-like; DRT, defense-associated reverse transcriptase; PD, phage defence; CRISPR, clustered regularly interspaced short palindromic repeats; Cas, CRISPR-associated protein, Stk, Serine/threonine kinase; SoFic, standalone protein with Fic domain.

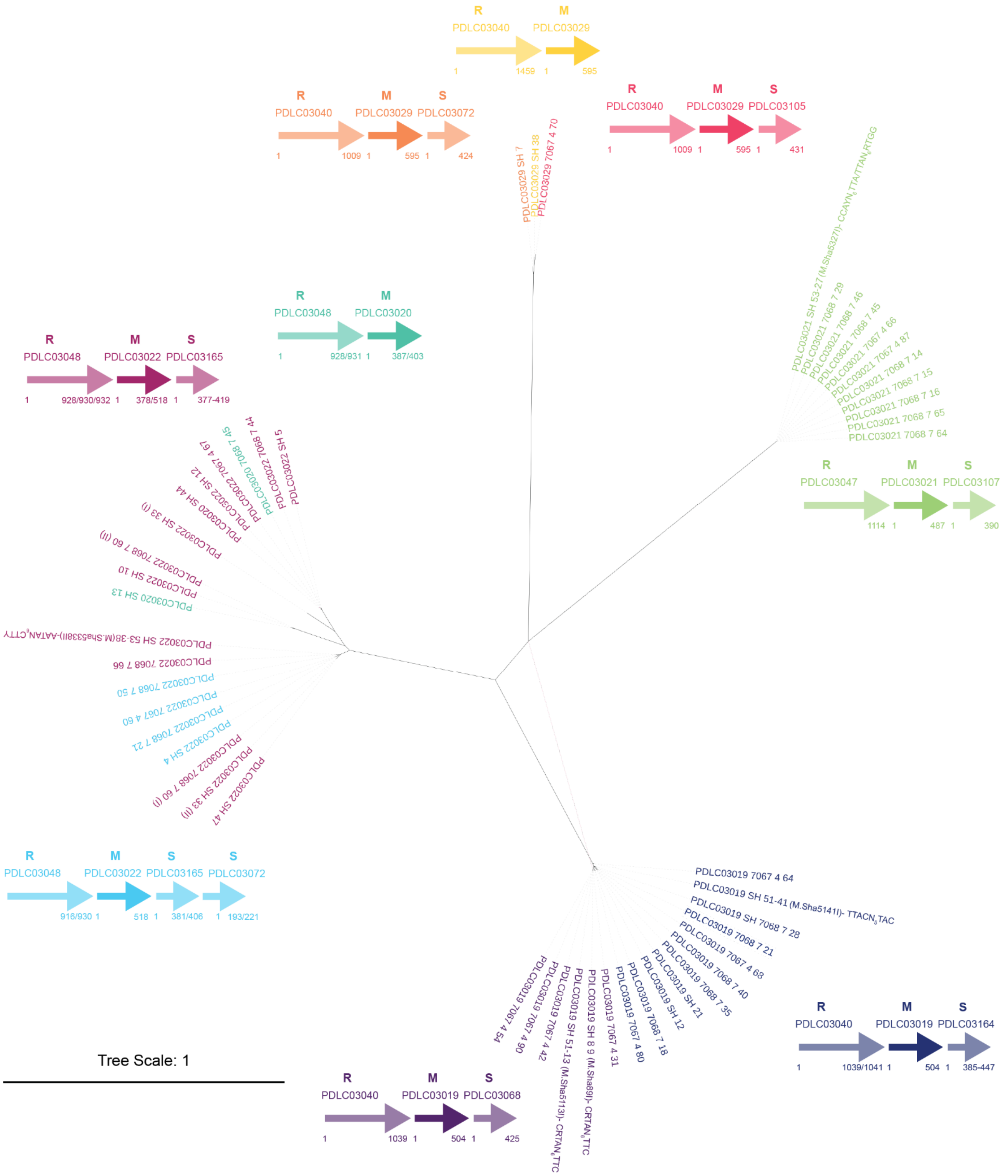

Figure S2A Type I MTases detected in *S. haemolyticus* genomes

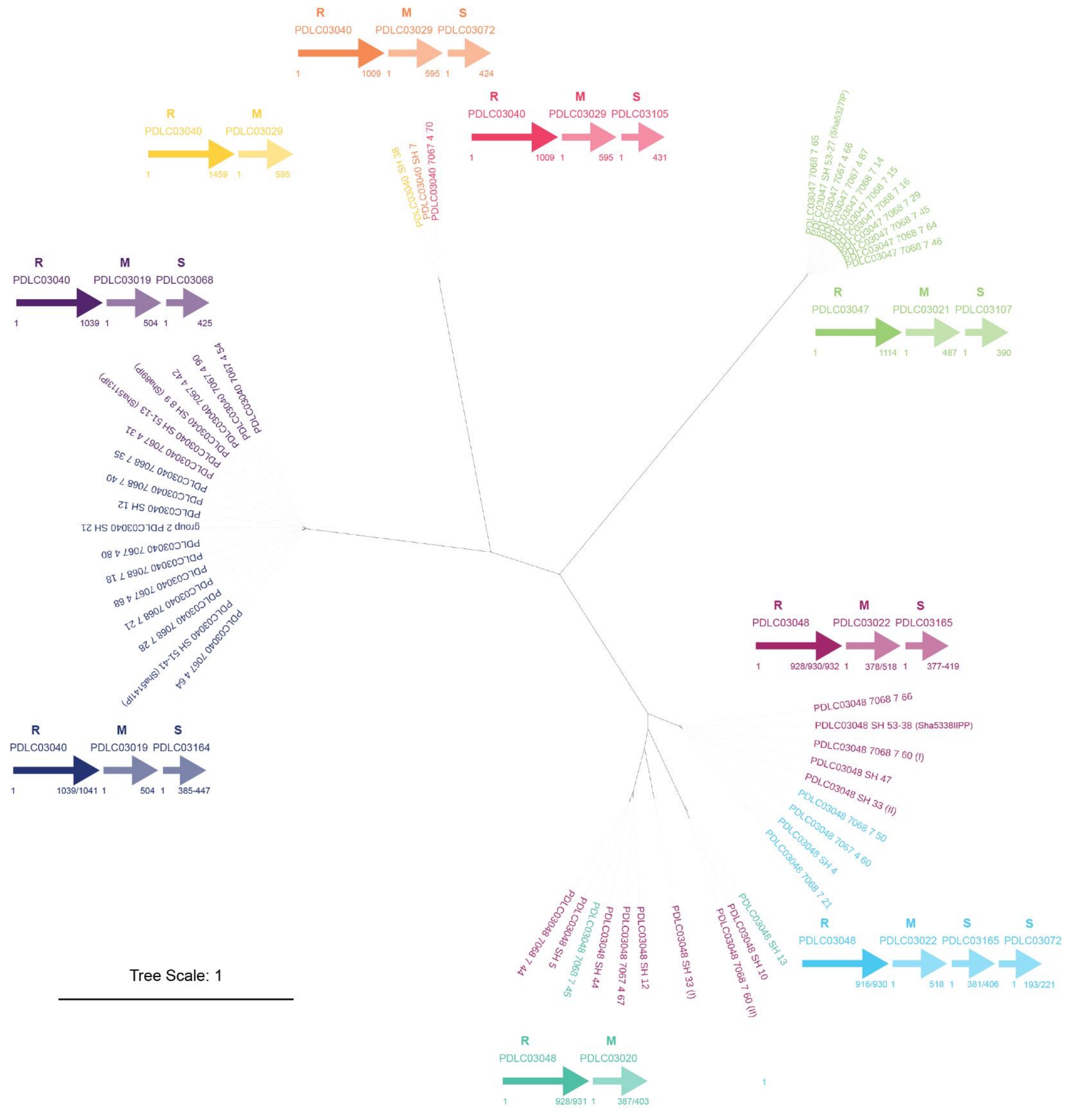

Figure S2B Type I REases detected in *S. haemolyticus* genomes

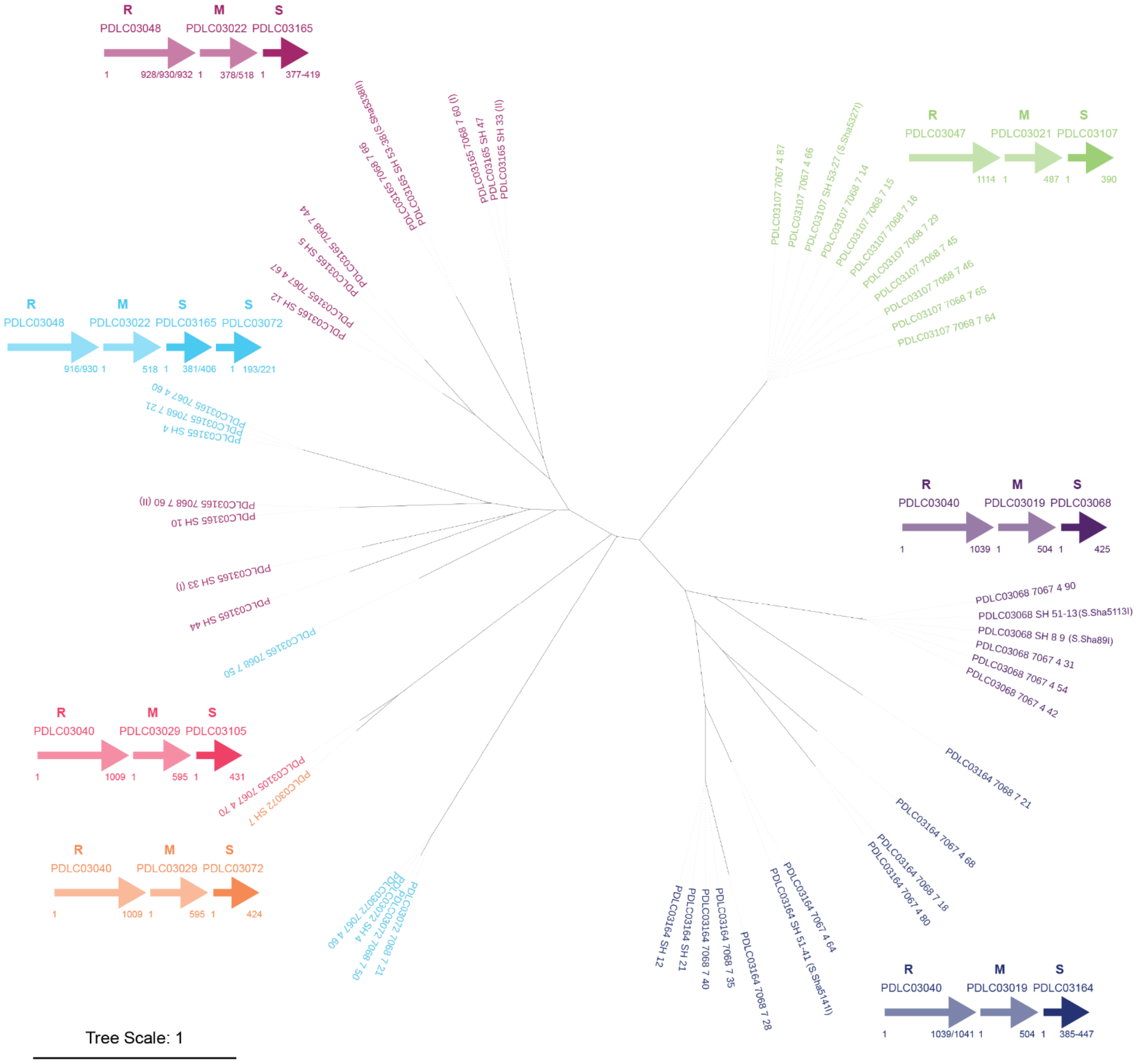

Figure S2C Type I Specificity subunits detected in *S. haemolyticus* genomes

Figure S2A-C) Maximum-likelihood dendrograms based on MUSCLE-aligned amino acid sequences of Type I system proteins identified by PADLOC. Branches represent proteins with highly similar sequences. Leaf labels show PADLOC accession number and the strain identifier for the genome in which the protein was detected. PADLOC and REBASE data was matched (Supplementary Table X) and, when available, the REBASE-assigned methylation motif is also included. Colours indicate proteins which occur together as part of the same system. The arrangement of the genes in each system is also given; bold arrows correspond to the elements present on the tree. R, restriction enzyme (REase), M, methyltransferase (MTase), S, specificity subunit.

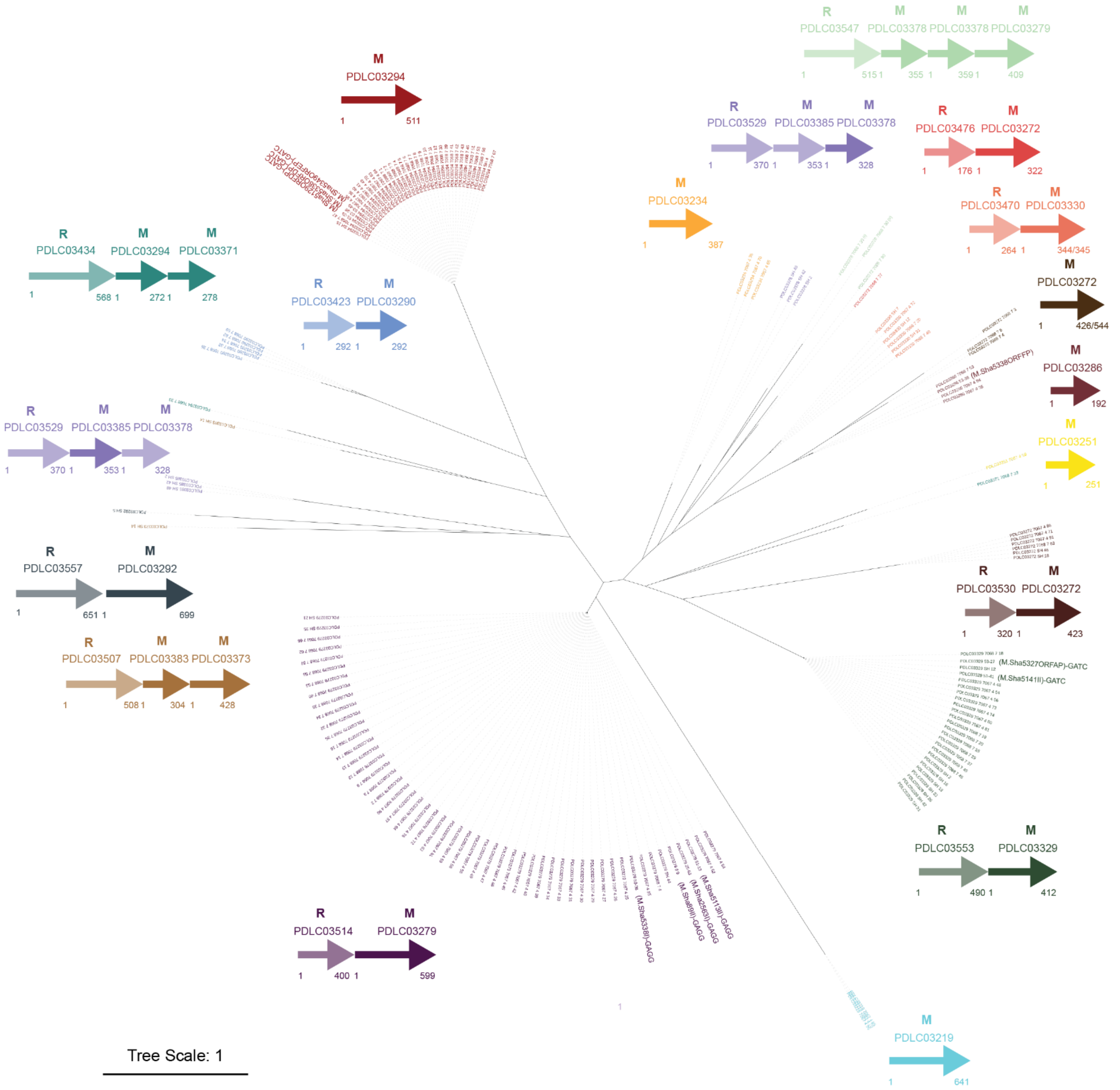

Figure S3A Type II MTases detected in *S. haemolyticus* genomes

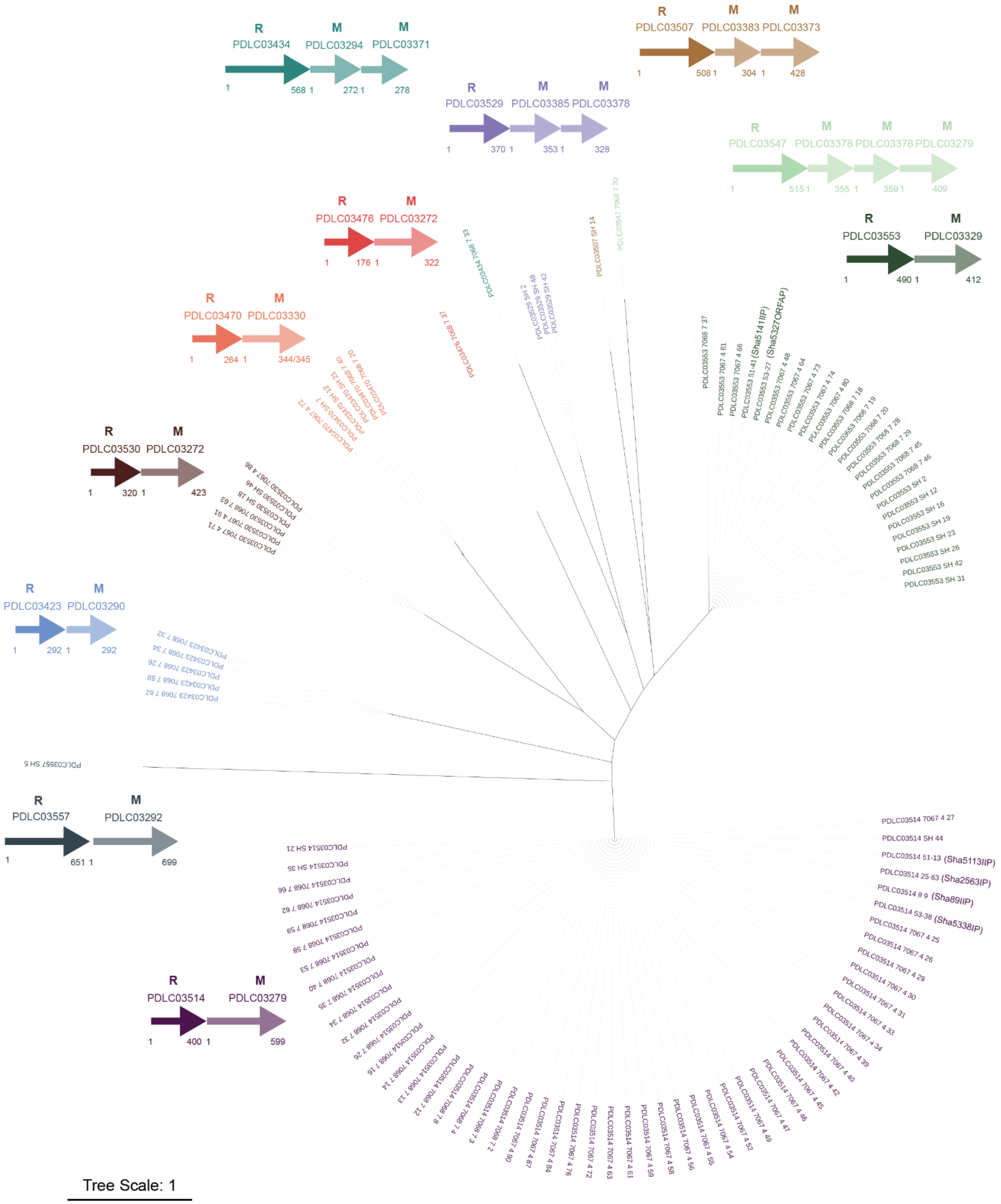

Figure S3B Type II REases detected in *S. haemolyticus* genomes

Figure S3A-B) Maximum-likelihood dendrograms based on MUSCLE-aligned amino acid sequences of Type II system proteins. Branches represent proteins with highly similar sequences. Leaf labels show PADLOC accession number and the strain identifier for the genome in which the protein was detected. PADLOC and REBASE data was matched (Supplementary Table X), and when available, the REBASE-assigned methylation motif is also included. Colours indicate proteins which occur together as part of the same system. The arrangement of the genes in each system is also given; bold arrows correspond to the elements present on the tree. R, restriction enzyme (Rease), M, Methyltransferase (MTase).

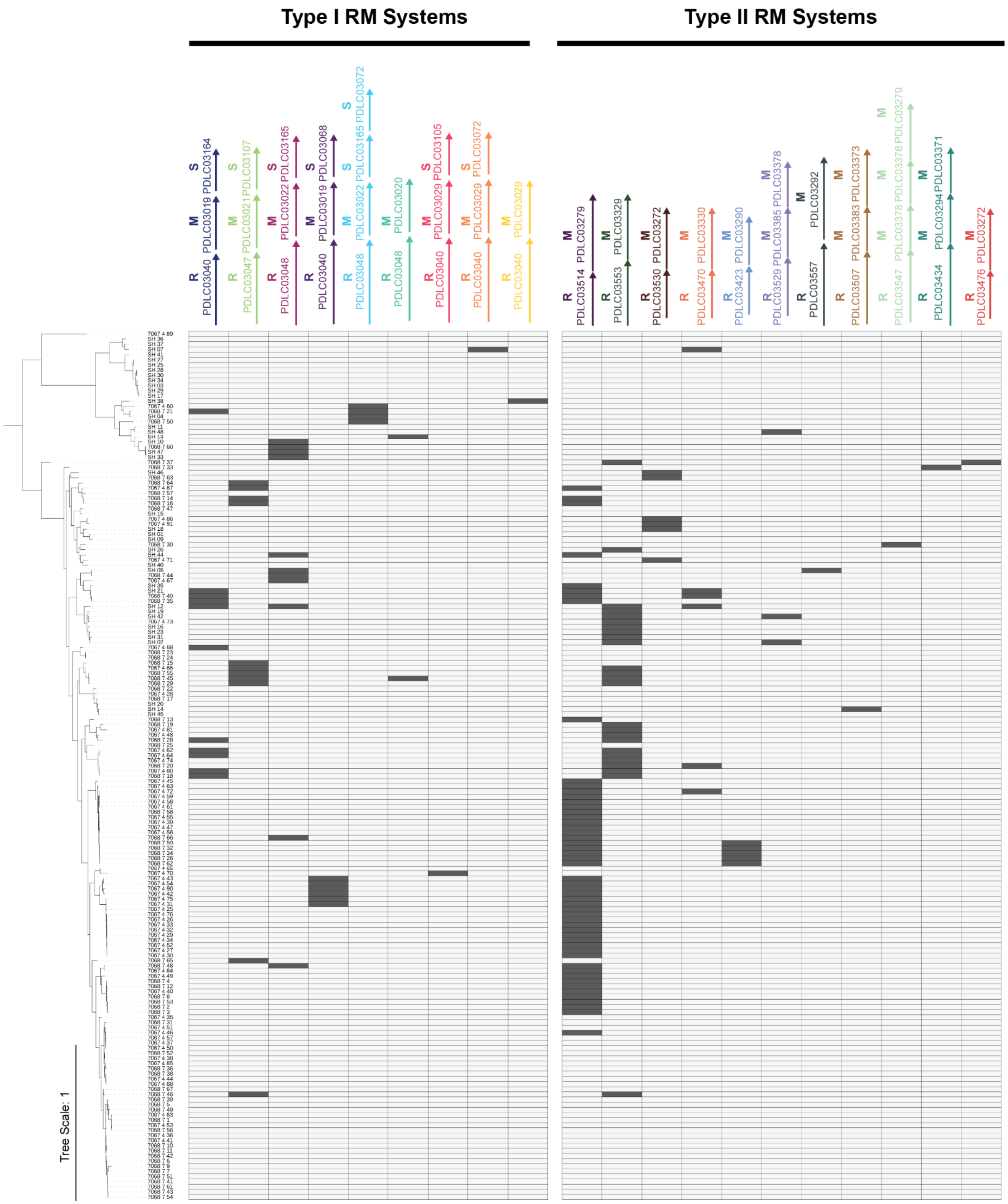

Figure S4) A reference-free phylogenetic tree constructed from whole genome sequences showing the distribution of Type I and Type II systems across *S. haemolyticus* isolates

Supplementary Table S1) Primers used in this study. Overlaps are underlined and bolded.

Primer name	Primer sequence (5'-3')	Characteristics/References
**Bisulfite sequencing**		
B1_CON_FW	TGTTTTGAATTATTTAATTATTTTGGT	Amplification of bisulfite-converted DNA
B1_CON_RV	ACACTTATAACACTTACACAC	Amplification of bisulfite-converted DNA
B2_CON_FW	GTATAATTATTAAAAATGTAAAGTTTGTT	Amplification of bisulfite-converted DNA
B2_CON_RV	CTTACAATATTAAATTTCTATAACACACAC	Amplification of bisulfite-converted DNA
B3_CON_FW	CACATATTAAAAACAAAAAAAATAAAATATATATC	Amplification of bisulfite-converted DNA
B3_CON_RV	GTTTTTATATAAATATTATGTTTTATAATTTTTATTTTAG	Amplification of bisulfite-converted DNA
B4_CON_FW	TGGTGTTTTGTGAGGTGATGT	Amplification of bisulfite-converted DNA
B4_CON_RV	AAAAAAATCATTAATTACTTAAATACTAAAAATCCTTTTA	Amplification of bisulfite-converted DNA
**Syngenic pEPSA5 (GAGG)**		
Syn_PartA_F	**GAGCTATTAAGCCGA**CCATTCGACAAGTTTTG	Overlap
Syn_PartA_R	CTGTTAGCACTTATATTTTGTTATTGTTCTTCTTCGATTTC	Overlap
Syn_PartB_F	**CAAAATATAAGTGCTAACAG**TCGTCTGCAAGTTTAG	Overlap
Syn_PartB_R	**CCCAGTTGGGG**TCGACTCTAG	Overlap
Vec_F	CCCCAACTGGGGTAACCTTTG	Overlap, vector linearization
Vec_R	TCGGCTTAATAGCTCACGCTATGC	Overlap, vector linearization
INS_vec_PartA_F	CTTTAAAATGCCCCTTAAAATTCAAAATAAAGGC	Plasmid insertion check
INS_PartAB_R	TACTAGATGTTGGCAAACGATATGAGC	Plasmid insertion check
**Assembly of DNA editing templates for recombineering (insertion of GAGG MTase)**		
Dcm_UP_FW	**AAATAAGGTTAACAT**TCGGTAAGCGCTTCATCCG	Overlap
Dcm_UP_RV	**TTTAAATT**CCATGGCCACTCGCAGCAAAAATATG	Overlap
Dcm_DOWN_FW	**GAATTTGATATTTAA**AGATTTCACCGGCCATCTG	Overlap
Dcm_DOWN_RV	**CGGGGATCCTGTCC**AGGATGCGGATCG	Overlap
MTase_FW	**GCGAGTGGCC**ATGGAATTTAAAATATTAGATTTG	Overlap
MTase_RV	**CCGGTGAAATCT**TTAAATATCAAATTCTAAGTTAG	Overlap
pRRS_Vector_FW	**CATCCTGGACAG**GATCCCCGGGGAAGATC	Overlap
pRRS_Vector_RV	**TGAAGCGCTTACCGA**ATGTTAACCTTATTTCCTGC	Overlap
Plas_ins_up_F	ATTAATGTGAGTTAGCTCACTCATTAGG	Plasmid insertion check

Plas_ins_down_R	AATACCGCACAGATGCGTAAG	Plasmid insertion check
pRRS_Dcm_51-13_ET_FW	TCGGTAAGCGCTTCATCCGTCAGC	Produces linear editing template
pRRS_Dcm_51-13_ET_RV	TGTCCAGGATGCGGATCGGCTG	Produces linear editing template
**Knockouts using pIMAY-Z**		
IM3-R	AATACCTGTGACGGAAGATCACTTCG	Insertion check. Monk and Stinear (1)
IM4-F	TACATGTCAAGAATAAACTGCCAAAGC	Insertion check. Monk and Stinear (1)
T3_FW	GCATGTAAAAAACAAGCGGGCTTTGC	TI target removal
T3_RV	CAATACAATGTAGGCTGCTCTACAC	TI target removal
SecA-1-R	**AACACCTCATT**ATTAGCCATTTTGATCACCTCG	Overlap
SecA-1-F	**ACAAAAGCTG**TACTACAACCTTTATGTATGGTACGAAATTACGCA	Overlap
SecA-2-F	**CAAAATGGCTAAT**AATGAGGTGTTCATAGATGACAATATA	Overlap
SecA-2-R	**GGGCGAATTGT**ATAATTTGGCACGATTCTCATATG	Overlap
SecA-VEC-F	**CCAAATTATACAA**TTCGCCCTATAGTGAGTCGT	Overlap, vector linearization
SecA-VEC-R	**GGTTGTA**GTACAGCTTTTGTTCCCTTTAGTG	Overlap, vector linearization
SecA-KOcheck-F	TTATCACCAGTATTGAGTCAACAAGG	Genome deletion check
SecA-KOcheck-R	GTACAACAATACCGACTTTACTTGATC	Genome deletion check
SraP_T1_FW	GAGGCTAAAGTATAATAATTGGTTGGAATAATGTATG	TI target removal
SraP_T1_RV	TTTTTTTAAGGATAGACAACCAATTTATAGAAAAG	TI target removal
SraP-1-F	**CAAAAGCTGG**TTTTTTGATTAATTTTTATTTTTC	Overlap
SraP-1-R	**GT**AATACTCTCCTTTATTCTCACACTTAC	Overlap
SraP-2-F	**GGAGAGTATT**ACTACTTCATGTGGGAGATAG	Overlap
Srap-2-R	**GCGAATTGG**TTATAATTCTTTCCTTATAATGC	Overlap
SraP-VEC-F	**GAATTATAAC**CAATTCGCCCTATAGTGAGTCG	Overlap, vector linearization
SraP-VEC-R	**CAAAAAAC**CAGCTTTTGTTCCCTTTAGTGAG	Overlap, vector linearization
SraP-KOcheck-F	GTTATGGCTACAACAATGAGGGAGAG	Genome deletion check
SraP-KOcheck-R	GTGTTGGTTATTAAATAATGATTTGATTAAACTTGTTAATAC	Genome deletion check
CapA_DW_FW	**CGAAATTAGAATTTG**CATGGCTAAAAATAAAAAAGAAGTAAG	Overlap
CapA_DW_RV	**GCGAATTG**CATCTGTCACAGTGTTTACTGGT	Overlap
CapA_UP_FW	**GAACAAAAGCTG**ATAAATGTAGAACCTTAAAGGAGTGAC	Overlap
CapA_UP_RV	**GCCATG**CAAATTCTAATTTCGATATTAATTTTATATTTTTAACATGG	Overlap

CapA_Vec_FW	**GACAGATG**CAATTCGCCCTATAGTGAGTCGTAT	Overlap, vector linearization
CapA_Vec_RV	**TTCTACATTTAT**CAGCTTTTGTTCCCTTTAGTGAGGG	Overlap, vector linearization
CapA_KO_Check_FW	GCTAATTCATCTAATGGGTGAGCTGTAATG	Genome insertion check
CapA_KO_Check_RV	CTTCCTTACTTTTAGGACCGTCATCTAC	Genome insertion check
CapA_T1_FW	CTCTGTGTAGAAATCTCTTGGCTTTTTTATATTTATG	TI target removal
CapA_T1_RV	ATATCTTGGCTTAAAACATTAATATTCAATATTTGGC	TI target removal
CapI_DW_FW*	**AAGGATAAAAAA**ATATAGTGGTGAGAAAATGAGTAAATCCAAAGATTT**C**ATATTTAG	Overlap, TI target removal
CapI_DW_RV	**GGCGAATTG**CTGAAAATATTGAAACATCATAGAAGTACAC	Overlap
CapI_UP_FW	**AACAAAAGCTG**GATAATGAAAAAAACTTCCTGTCACTAATGAATGC	Overlap
CapI_UP_RV	**CTCACCACTAT**ATTTTTTTATCCTTTCTTTACACTATTCATCAAAG	Overlap
CapI_Vec_FW	**CAATATTTTCAG**CAATTCGCCCTATAGTGAGTCGTATTACG	Overlap
CapI_Vec_RV	**TTTTCATTATC**CAGCTTTTGTTCCCTTTAGTGAGG	Overlap
CapI_KO_Check_FW	CACGGAACATAGTAGACGCTATTTATTAGATG	Genome insertion check
CapI_KO_Check_RV	CATCATTTACTTCGTAGCTTAAAAGTACTGTTAC	Genome insertion check
Epsn_INS_FW	GTGATTGAATGTGTAAAACTTTTTTATCTGTTAAAAG	Overlap
Epsn_INS_RV	CGTTGATGACCGTGATAAGAAAGAC	Overlap
Epsn_DW_FW	**AATCTTAACTAA**ATGAAAGCACTTTGTCGATGATATAAATC	Overlap
Epsn_DW_RV	**GGGCGAATTG**TTATCTAAACTAGAAAAGAATGATAAAGGGTTCTTCTTAATGG	Overlap
Epsn_MID_FW	**GCAAGTAGTTTATTTATTACATATTTAC**ATGATAGTTACATTAGTTTTAACGTAAATTCTAAC	Overlap
Epsn_MID_RV	**AAGTGCTTTCAT**TTAGTTAAGATTTAAGTTTTCTTTTAATTTCTCATTAACT	Overlap
Epsn_UP_FW	**CAAAAGCTG**CATCTAATGGGTGAGCTGTAATGTATAATAATTTTGC	Overlap
Epsn_UP_RV	**GTAACTATCATGTAAATATGTAATAAATAAACTACTTGC**GTAATGAATTAGTAGC	Overlap
Epsn_vec_FW	**GTTTAGATAA**CAATTCGCCCTATAGTGAGTCGT	Overlap, vector linearization
Epsn_vec_RV	**CCATTAG**ATGCAGCTTTTGTTCCCTTTAGTGAGGG	Overlap, vector linearization

Supplementary Table S2) Plasmids used in this study

Plasmid	Addgene number	Reference
pRRS		Johnston, Cotton (2)
pEPSA5		Johnston, Cotton (2)
pTarget	#62226, modified by Johnston et al. for *Dcm* target	Jiang, Chen (3), Johnston, Cotton (2)
pCas	#42876	Jiang, Chen (3)
pIMAY-Z		Monk and Stinear (1)

Supplementary Table S3) Bacteria strains used in this study

Strain/organism	Description/Accession number	Reference
S. haemolyticus 8-9	SAMEA1035041	Cavanagh, Klingenberg (4)
S. haemolyticus 25-63	SAMEA1035076	Cavanagh, Klingenberg (4)
S. haemolyticus 51-13	SAMEA1035044	Cavanagh, Klingenberg (4)
S. haemolyticus 51-29	SAMEA1035047	Cavanagh, Klingenberg (4)
S. haemolyticus 51-41	SAMEA1035075	Cavanagh, Klingenberg (4)
S. haemolyticus 53-27	SAMEA1035140	Cavanagh, Klingenberg (4)
S. haemolyticus 53-38	SAMEA1035061	Cavanagh, Klingenberg (4)
S. haemolyticus 53-49	SAMEA1035106	Cavanagh, Klingenberg (4)
S. haemolyticus 57-1	SAMEA5568753	Cavanagh, Klingenberg (4)
S. haemolyticus 51-22	SAMEA5568761	Cavanagh, Klingenberg (4)
E.coli JMC1	Minicircle producing *E. coli*	Johnston, Cotton (2)
E.coli JMC4	Minicircle producing *E. coli* mimicking *S. haemolyticus* MTase.	This study

References:

1. Monk IR, Stinear TP. From cloning to mutant in 5 days: rapid allelic exchange in Staphylococcus aureus. Access Microbiology. 2021;3(2).
2. Johnston CD, Cotton SL, Rittling SR, Starr JR, Borisy GG, Dewhirst FE, et al. Systematic evasion of the restriction-modification barrier in bacteria. Proceedings of the National Academy of Sciences. 2019;116(23):11454-9.
3. Jiang Y, Chen B, Duan C, Sun B, Yang J, Yang S. Multigene editing in the Escherichia coli genome via the CRISPR-Cas9 system. Appl Environ Microbiol. 2015;81(7):2506-14.
4. Cavanagh JP, Klingenberg C, Hanssen AM, Fredheim EA, Francois P, Schrenzel J, et al. Core genome conservation of Staphylococcus haemolyticus limits sequence based population structure analysis. J Microbiol Methods. 2012;89(3):159-66.
